# Compulsory Service in the R.A.M.C.

**Published:** 1916-09-02

**Authors:** 


### COMPULSORY SERVICE IN THE R.A.M.C.

### How the Profession has Avoided Direct Governmental Control.

Absorbed as everyone is in the fume and fret of this cataclysmic war, it is not easy to dwell anywhere but in the present and in the future, or to gain sufficient leisure and detachment to review the past. Yet some such survey of the evolution of the relation of the medical profession to the State during these two years of change and pressure must be attempted if any estimate worth having is to be formed of how those relations are likely to develop in the future. Both those who have recently entered the ranks of the profession and those who hope some day to do so are vitally concerned in any radical change that may take place in the position of medicine in the body politic; and though to forecast the future is impossible, at least it is feasible to examine current tendencies as impartially as imperfect knowledge and inescapable prejudice will allow.

The needs of the Army (and Navy) for medical officers have, during the last twelve months, begun definitely to exceed the supplies available by voluntary enlistment. This plain statement of an indisputable fact carries the inevitable corollary of State interference to supply those needs, and it is to the credit of the medical profession that that corollary was accepted and faced at a time—about a year ago—when politicians were still hesitating and lobbying over the similar failure of voluntary recruiting to fill the cadres of the New Armies. The result of this appreciation of the stern logic of events by the profession as a whole was that preparation was begun whereby the application of the principle of compulsion might be made with less injustice, less inefficiency, less lack of insight and sympathy than any department of Government officials (civil or military) could be expected to exercise. In other words, the profession for once in a way showed itself equal to a social and political emergency, and has so far surmounted its problems with considerable success and a minimum of internal dissension.

### The Medical Profession and State Control.

This achievement is in itself so remarkable, especially when the story of the political helplessness of the medical profession is borne in mind, that one might expect to find at the root of it some motive of fairly general acceptance. The foundation of the unity and singleness of purpose which the profession has displayed in this connection during the last year is, we believe, dislike and distrust of the idea of a State Medical Service. Whether this antipathy is well or ill founded is a large question, and one to be looked at from several points of view; at any rate, we do not propose to discuss it here. But if our diagnosis of the situation is correct, and certain proceedings at the recent annual representative meeting of the British Medical Association strongly confirm it, it is this hatred of direct State control that has largely actuated the rank and file of the medical profession and produced the extraordinary unanimity (comparatively speaking) on the question of the arrangements for filling the commissioned ranks of the Royal Army Medical Corps. The guardians, therefore, of any young men or women who are thinking of entering the medical profession in the near future may, if they want to get a longer view of the prospects than most young people will take of their own accord, note that any radical upheaval of the existing individualistic system in the profession along advanced socialistic lines is certain to be most strongly opposed by those who at present would be most directly affected, and is to that extent an unlikely possibility for some years to come.

The exact steps by which the apprehension of anything which might conduce to direct State control led to the present situation in the profession are worth recapitulating, if merely to show that the profession can act when once it can be induced to make up its mind on any serious topic; the insuperable difficulty, as a rule, is to arouse sufficient interest in any ethico-political question to get the mind of the profession made up.

### The Central Medical War Committee.

A year ago, then, the failure of normal voluntary recruitment of medical officers was, if not imminent, at least clearly visible upon the horizon to instructed eyes. At that time, though Registration had been carried into effect, the nation's rulers had not by any means abandoned the voluntary system for the country at large, and had hardly dared to admit that compulsion might come; though a constantly increasing section of all the loyalest, sanest, and most clear-headed of the population already regarded some form of conscription as imperative for the safety of the commonwealth. The famous Derby recruiting campaign was shortly to be inaugurated; and whether it should succeed or should fail in postponing compulsion, in either event the medical profession stood to be called upon to provide shortly a much increased contingent of medical officers.

In these circumstances a self-elected committee arose in an endeavour to meet the situation. It is true that the British Medical Association had the largest share in the promotion of this committee; but officially there was no connection between the two, and it is not inaccurate to say that the committee elected itself. Whatever its origin, the Central Medical War Committee, as it was called, showed a great deal of activity; and that activity was principally directed towards retaining in its own control the supply of doctors demanded from civilian practice by the military authorities. Voluntary enlistment the committee did its utmost to promote, and by driving a series of bargains with the War Office it prepared the way for making itself indispensable in the event of compulsion coming to pass. Though the composition of the committee was criticised at times, and though certain of its actions excited a good deal of resentment among sections of the medical profession, on the whole it can safely be said that it steered a judicious course through troubled waters and has distinctly deserved well of the profession. The most influential—indeed, so far as we know, the only serious—attempt to undermine its position took place early this year. The malcontents had, as a matter of fact, certain grounds for legitimate criticism; fortunately they were met in a most conciliatory spirit and were soon convinced that the broad lines of the committee's work were sound and worthy of support.

What the Committee has done for the Doctors.

The result of steady spade work all through the winter, and of the collapse of the only vocal opposition just mentioned, was that by the time National Service was at last adopted by Parliament, the Central Committee was in an exceedingly strong position. There was, probably enough, no very pressing desire at the War Office or in the Government to undertake the regimentation of the medical profession, so long as the requirements of the Navy and Army were duly met. Luckily for the doctors, they are indispensable alike to the civilian population and to the Forces; so that it would in any case be impossible to make a clean sweep of them (of those, that is, within the military age limit) as can be done with many other professions. This advantage enabled the Central Committee, in return for taking over a responsibility which would in any case have been extremely onerous and difficult for Government officials, to extract, as consideration for its help, various concessions for the profession, of which the most important and most appreciated is that permitting enlistment for periods of twelve months at a time only, instead of for the duration of the war. Then by skilfully displaying these concessions, and by hints that recalcitrance would mean their withdrawal, the committee (even before the adoption of National Service immensely strengthened its hand) long ago secured the adhesion to its schemes of the great bulk of the profession, thus making its position doubly secure with the War Office. A salutary circle was thus established; the committee used its influence with the medical profession to make itself constantly more and more useful to the War Office, and its usefulness at the War Office to increase as regularly its prestige with the members of the profession.

How the Past Illumines the Future.

This retrospect of the last twelve months has a definite bearing on the future of the medical profession. It shows that the profession can and does adapt itself, of its own free will and accord, to meet the proven necessities of the State in a grave national crisis. It does possess, what acute observers at the time of the National Insurance Act were disposed to doubt, a consciousness of corporate responsibility, a spirit and soul of its own. It shows, moreover, that detestation of a State Medical Service—or rather of anything that may in the least further such a future development—is a more powerful lever in awaking that corporate spirit than was the discontent aroused by the Insurance Act proposals. It has shown the profession the benefits of organisation and the strength of its own position, two things which many of its leaders have often despaired of ever drumming into the heads of its members. It has, therefore, made the introduction of a State Medical Service impossible without the previous consent of a great majority of the medical men of the day. At present the temper of the profession in Britain, if we read it aright, is very much against any such surrender to State control. But it must not be forgotten that after this war almost the whole of the doctors qualified this century will have themselves served the State as whole-time salaried officers, and that their views about such service may not at all coincide with the prepossessions of those at home of the older generation. As to that, all prophecy is futile; but the possibility is one to be kept in mind by anyone who may try to visualise the state of the medical profession fifteen or twenty years hence. Such a prevision may not unnaturally engage the attention of parents or guardians with boys or girls to "place" in a profession; and considerations relevant to an attempt at it are, in consequence, not perhaps out of place in the Educational Number of The Hospital.

